# Effects of Face and Background Color on Facial Expression Perception

**DOI:** 10.3389/fpsyg.2018.01012

**Published:** 2018-06-21

**Authors:** Tetsuto Minami, Kae Nakajima, Shigeki Nakauchi

**Affiliations:** ^1^Electronics-Inspired Interdisciplinary Research Institute, Toyohashi University of Technology, Toyohashi, Japan; ^2^Department of Computer Science and Engineering, Toyohashi University of Technology, Toyohashi, Japan; ^3^Center for Information and Neural Networks, National Institute of Information and Communications Technology, Osaka University, Hyogo, Japan

**Keywords:** facial color, facial expression recognition, angry faces, fearful faces, psychometric function, point of subjective equality, reaction times

## Abstract

Detecting others’ emotional states from their faces is an essential component of successful social interaction. However, the ability to perceive emotional expressions is reported to be modulated by a number of factors. We have previously found that facial color modulates the judgment of facial expression, while another study has shown that background color plays a modulatory role. Therefore, in this study, we directly compared the effects of face and background color on facial expression judgment within a single experiment. Fear-to-anger morphed faces were presented in face and background color conditions. Our results showed that judgments of facial expressions was influenced by both face and background color. However, facial color effects were significantly greater than background color effects, although the color saturation of faces was lower compared to background colors. These results suggest that facial color is intimately related to the judgment of facial expression, over and above the influence of simple color.

## Introduction

As humans, we exhibit facial color changes such as blushing and paleness, and physiological studies have demonstrated that these changes are associated with specific emotional states. The face flushes during anger (Darwin, 1872; Drummond and Quah, 2001; Montoya et al., 2005) or pleasure (Drummond, 1994), and turns pale while feeling fear (Drummond, 1997; Montoya et al., 2005).

Facial color is an important cue in detecting others’ emotional states (Palmer and Schloss, 2010), and as such, is a core component of social interaction. However, facial color is also essential for face perception (Sinha, 2002; Vuong et al., 2005; Nederhouser et al., 2007; Russell et al., 2007), and modulates facial recognition at the behavioral level (Yip and Sinha, 2002; Balas et al., 2010). At the same time, facial color affects a range of social judgments such as health, sexual signaling, and emotion (Thorstenson, 2018). Among them, emotional perception is of importance for our daily life.

We have previously demonstrated that facial color clearly influences the judgment of facial expression using morphed facial expressions as experimental stimuli (Nakajima et al., 2017). However, color itself is also known to convey meaningful information such as attractiveness (Elliot and Niesta, 2008; Guéguen, 2012; Pazda et al., 2013; Young, 2015). In addition, previous studies have reported that a red background color to presented faces enhances the perception of anger, or otherwise negative expressions (Young et al., 2013; Gil and Le Bigot, 2015).

Thus, both facial color and background color affect the perception of facial expression, but it is unknown which makes the most substantial contribution. Red is interpreted as a signal of danger in competition contexts (Elliot et al., 2007). Socially anxious people exhibited negative bias, in which they tend to interpret neural faces as negative expressions (Yoon and Zinbarg, 2008). So the background color might bias the processing of anger expressions in a top-down manner. On the other hand, a facial color has the similar effect as the background color, but facial color is recognized as anger associated with increased blood flow. That is, anger perception is promoted in a bottom-up manner compared with the background color.

The goal of our study was to examine whether facial color or background color is more effective in the judgment of facial expression. On the basis of our previous findings we predicted that the effects of facial color would be significantly greater than those of background color. In the comparison of these effects, we mainly analyzed the PSE (Point of subjective equality at 50% performance) and reaction times (RTs) to facial expression. However, judging ambiguous stimuli is known to be sensitive to response bias. For example, the mood of participants affects facial expression cognition (Schmid and Schmid Mast, 2010). Therefore, we also analyzed response bias using signal detection theory and investigated whether these scores depend on a variety of colors.

## Materials and Methods

### Participants

Twelve healthy participants (of which 6 were females) participated in this experiment (mean age = 23.25 years, *SD* = 1.36). According to self-report, none of the participants had color blindness. All participants provided written informed consent. The experimental procedures were approved by the Committee for Human Research at the Toyohashi University of Technology, and the experiment was conducted in accordance with the guidelines of the committee.

### Stimuli

**FIGURE 1:**
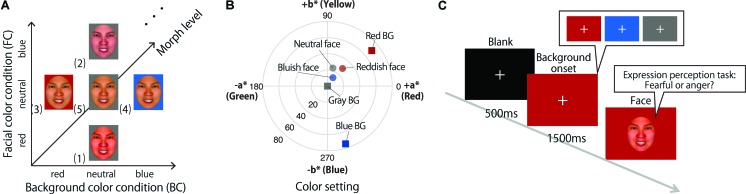
Stimuli and procedure: **(A)** facial color (FC) and background color (BC) conditions. (1) A reddish-colored face with a gray background; (2) a bluish-colored face with a gray background; (3) a neutral-colored face with a red background; (4) a neutral-colored face with a blue background; and (5) a neutral-colored face with a gray background. **(B)** Each stimulus on CIELab color space. **(C)** Experimental paradigm. Each trial began with a fixation for 500 ms, followed by a colored background (red, blue, or gray) for 1,500 ms, and then an expression morphed face was presented until participants responded.

Color images of emotional faces (four females and four males posing as fearful and angry) were taken from the ATR Facial Expression Image Database (DB99) (ATR-Promotions, Kyoto, Japan), as used in Nakajima et al. (2017). The database has ten different types of facial expression from four female and six male Asian models. The database also includes the results of a psychological evaluation experiment (unpublished data) that tested the validity of the database. External features (i.e., neck, ears, and hairline) were removed from the face images using Photoshop CS2 (Adobe Systems Inc., San Jose, CA, United States). We created three different colored faces by manipulating the CIELab a^∗^ (red-green) or b^∗^ (yellow-blue) values of their skin area as shown in **Figure 1A**. There were three facial color conditions, reddish-colored (+12 units of a^∗^), bluish-colored (-12 units of b^∗^), and natural-colored (not manipulated). In addition, we created three different-colored backgrounds: red (44.2/74.0/39.2), blue (44.2/74.7/287.6), and gray (44.5/–/265.7) in the CIE LCh color space as shown in **Figure 1A**. Facial expression continua were created by morphing between two different expressions for one identity of the same facial color condition in ten equal steps using SmartMorph software (MeeSoft^1^ as shown in **Figure 1B**. Fear-to-anger expressions were selected for morphing because the effect of facial color on expression was found to be the strongest on these previously (Nakajima et al., 2017). In total, 264 images were used in this experiment [3 facial colors × 8 models (4 females) × 11 morph increments]. All face images were 219 pixels × 243 pixels (11.0° × 12.2° visual angle). Images were normalized for mean luminance and root mean square contrast.

### Procedure

The experiment was performed in four blocks with the following five conditions: (1) a reddish-colored face with a gray background; (2) a bluish-colored face with a gray background; (3) a neutral-colored face with a red background; (4) a neutral-colored face with a blue background; and (5) a neutral-colored face with a gray background. We set (1) and (2) to check the effect of facial color, (3) and (4) to check the effect of background color, and (5) as a control condition (**Figure 1A**). Each trial began with a fixation point for 500 ms, followed by a colored background (red, blue, or gray) for 1,500 ms, and then an expression morphed face was presented until participants responded (**Figure 1C**). Participants were requested to identify the expression of the face regardless of facial color as quickly and accurately as possible by pressing one of two buttons. Each face was presented in a random order, resulting in a total of 220 trials (5 conditions × 11 morph increments × 4 models) per block.

### Analysis

The expression perception rate and mean response times were computed for each morphed face. The expression perception rate from each participant was fit with a psychometric function using a generalized linear model with a binomial distribution in Matlab software (MathWorks, Natick, MA, United States). To compare facial and background color effects on facial expression perception, the PSE (Point of subjective equality at 50% performance) was identified by finding the morph increment for each participant. The PSE differences from the control condition were calculated for each facial and background color conditions as a Color Effect Index (CEI). The CEIs were analyzed in a 2 (facial color or background color) × 2 (color: red, blue) repeated-measures ANOVA.

Besides, response biases were computed based on the assumption that signals arose from two categories: targets (anger) and non-targets (not-anger) and analyzed using analyzed in a 2 (facial color or background color) × 2 (color: red, blue) repeated-measures ANOVA.

To compare to the results of Young et al. (2013), we computed mean RTs in each condition for the 100% fearful and anger face expressions. The RT difference from control (FC: neutral-colored face, BG: gray background) were analyzed in a 2 (condition: FC and BG) × 2 (color: red and blue) × 2 (expression: 100% fearful and 100% anger) repeated-measures ANOVAs for each facial and background color condition.

## Results

**FIGURE 2:**
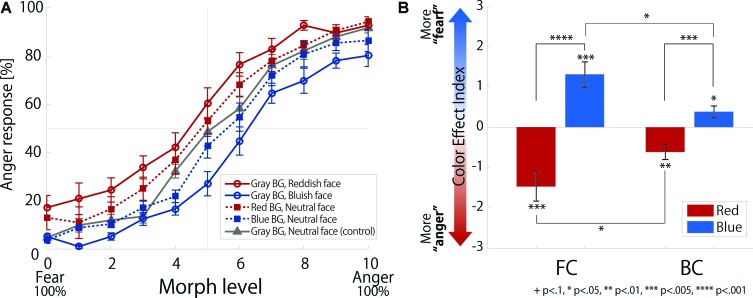
Psychometric function and color effect index. **(A)** The relation between morph level (fear-neural-anger) and response (percentage of anger response) to each stimulus. To compare effects on the facial expression perception, the PSE (Point of subjective equality at 50% performance) was identified by finding the morph increment for each participant. **(B)** Color Effect Index (CEI) of two colors in each condition. CEIs were calculated from PSE differences from the control condition.

**Figure 2A** shows the relation between morph level (fear-neutral-anger) and response (percentage of anger responses) to each stimulus. To compare effects on facial expression perception, we calculated a CEI based on the PSE for each participant (**Figure 2B**).

### Color Effect Index

The ANOVA showed a significant main effect of color [*F*(1,11) = 21.59, *p* < 0.001, $\eta_{p}^{2}$ = 0.66] and a significant interaction between condition and color [*F*(1,11) = 8.55, *p* = 0.014, $\eta_{p}^{2}$ = 0.44], but the main effect of condition was not significant [*F*(1,11) = 0.12, *p* = 0.739, $\eta_{p}^{2}$ = 0.01]. Subsequent analysis indicated that the CEI was significantly smaller for reddish faces but larger for bluish faces in the FC condition than in the BG condition [*F*(1,11) = 7.82, *p* = 0.017, $\eta_{p}^{2}$ = 0.42; *F*(1,11) = 7.27, *p* = 0.021, $\eta_{p}^{2}$ = 0.40]. This suggests that color effects in the FC conditions were significantly greater than those in the BG conditions.

### Response Biases

The ANOVA analysis showed no significant effect of facial color for response bias [*F*(1.11,12.17) = 1.00, *p* = 0.347, $\eta_{p}^{2}$ = 0.08). With respect to the BG condition, the ANOVA analysis showed the significant main effect of color for response bias [*F*(2,22) = 6.84, *p* = 0.005, $\eta_{p}^{2}$ = 0.38]. Subsequent analysis indicated the conservative tendency (with fearful response) to bluish background compared with other backgrounds (*p*s < 0.05).

### Reaction Times

**FIGURE 3:**
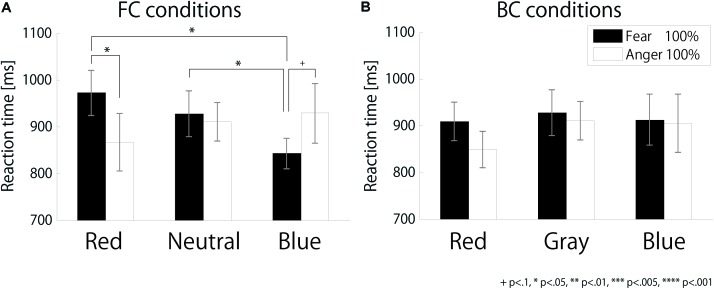
Reaction times to each color and expression (morph level 100%). **(A)** FC and **(B)** BC conditions.

**Figure 3** shows the RTs depending on color and expression in each condition [FC(a) and BG(b)]. The RTs were longer than those of a previous study (Young et al., 2013) because we used facial stimuli with 11 levels morphed expression while two extreme expressions were used in the previous study. Repeated-measures ANOVA showed a significant interaction between color and expression [*F*(1,11) = 39.77, *p* < 0.001, $\eta_{p}^{2}$ = 0.78]. There was no other main effect or interaction [condition (1, 11) = 0.25, *p* = 0.627, $\eta_{p}^{2}$ = 0.02; color: *F*(1,11) = 0.01, *p* = 0.908, $\eta_{p}^{2}$ < 0.01; expression: *F*(1,11) = 0.03, *p* = 0.867, $\eta_{p}^{2}$ < 0.01; interaction between condition and color: *F*(1,11) = 1.71, *p* = 0.218, $\eta_{p}^{2}$ = 0.13; interaction among the three factors: *F*(1,11) = 3.59, *p* = 0.085, $\eta_{p}^{2}$ = 0.25]. Subsequent analysis indicated that RTs were significantly shorter to red angry faces than to red fearful faces. [*F*(1,11) = 5.28, *p* = 0.042, $\eta_{p}^{2}$ = 0.32], not to blue faces [*F*(1,11) = 2.81, *p* = 0.122, $\eta_{p}^{2}$ = 0.20], which suggests that red color, irrespective of condition, shortened the RT to angry faces.

In addition, we analyzed the RTs separately in the FC and BG conditions. In the FC condition, there was a significant interaction between color and expression [*F*(2,22) = 7.19, *p* = 0.004, $\eta_{p}^{2}$ = 0.40]. Subsequent analysis showed that RTs were shorter for red angry faces than for red fearful faces [*F*(1,11) = 8.45, *p* = 0.014, $\eta_{p}^{2}$ = 0.43]. There was no other main effect [color: *F*(2,22) = 1.30, *p* = 0.293, $\eta_{p}^{2}$ = 0.11; expression: *F*(1,11) = 0.34, *p* = 0.573, $\eta_{p}^{2}$ = 0.03]. There was also no main effect or interaction in the BG condition [color: *F*(2,22) = 1.08, *p* = 0.358, $\eta_{p}^{2}$ = 0.09; expression: *F*(1,11) = 1.71, *p* = 0.218, $\eta_{p}^{2}$ = 0.13; interaction: *F*(2,22) = 2.08, *p* = 0.149, $\eta_{p}^{2}$ = 0.16].

## Discussion

In this study, we investigated behavioral performance in facial expression judgment by comparing a facial color condition (reddish-colored, bluish-colored, or natural-colored, i.e., not manipulated) with the background condition (red, blue, or gray). To compare the effects between facial and background colors on PSE, we defined the CEI. The reddish-colored faces or red BG were more likely to be identified as an anger expression, while those of bluish-colored faces or blue BG were more likely to be identified as a fear expression, consistent with previous results (Nakajima et al., 2017). The amplitude of the CEI on the face was significantly larger than that on the background, which suggests that facial color effects were significantly greater than those in the background condition, although the color saturation of the faces was lower compared to that of background color. Additionally, we compared RTs between the two conditions. On the whole, red color both on the face and as the background accelerated the judgment of anger faces. However, when we conducted a separate analysis in each condition, this effect was only found in the face condition. In summary, the effect of facial color was significantly larger than that of background color in expression judgment, although the impact of color difference on facial color was larger than that of background color.

What factors may produce these differences between facial and background color on the judgment of emotional expression? One possibility is that color perception for faces is enhanced compared to other things. Some previous studies suggest that color perception is enhanced for faces (Tan and Stephen, 2013; Thorstenson et al., 2017b). That is, color on the face looks redder, and the effect of color is enhanced. This predicts that the effects of facial and background color have the same function but at a different intensity. However, we set the hue of the background color to be considerably larger than that of facial color, and thus it is doubtful that the enhancement by facial color is due to difference in hue between the two conditions in this study.

Another potential explanation may be the specificity of facial color. The effect of color, such as background color, is subject to environmental factors such as culture. For example, recent studies have found that responses to color may vary depending on age, gender, culture, and preference (Ou et al., 2004; Manav, 2007). However, facial color has innate features. Anger and red are supposed to have strong connections due to the fact that anger produces an increase in blood flow on the face and neck (Drummond, 1997; Changizi et al., 2006). In addition, from an ecological point of view, Changizi et al. (2006) argue that color vision in primates has evolved for discriminating skin color modulations, such as in perceiving facial expression and assessment of health condition. Also, in the background condition, there was a significant difference in response bias, assumed to reflect top-down processes, which means that to a bluish background, a tendency of conservative judgment (not anger) was observed. Response bias to emotional stimuli such as facial expression and emotional words is a well-known phenomenon (Yoon and Zinbarg, 2008). This bias suggests that the effect of background color to facial expression is mediated via top-down processes. In addition, a previous study (Thorstenson et al., 2017a) found that the direction of emotion-color associations applied between faces and shapes was consistent, but that its magnitude was different by comparing the coloration changes of faces and shapes. They concluded that participants relied on abstract associations to emotion-color associations in shapes. Taken together, facial color likely has different functions on expression judgment compared to other factors, such as background color.

This study presents some limitations. A limitation is that our sample size is small. Another limitation of our study may be that we only presented Japanese faces to Japanese young participants and our findings may be subject to a race effect. Tan and Stephen (2013) reported a race effect in facial color perception, in which Asian participants performed better in response to Asian and Caucasian faces than to African faces. On the other hand, the properties of skin spectral reflectance do not differ among all races (Changizi, 2009), which suggests that the effects of facial color as shown in our study might be common within the same races. Our results might also be subject also to an age effect. For example, some previous studies have suggested that a negativity bias to emotional faces decays with the advance of age (Mather and Carstensen, 2003; Carstensen and DeLiema, 2018). Further studies are needed in order to investigate the effect of age and race on the facial expression judgment.

In summary, we found that facial color makes a greater contribution to expression judgment than background color. We suggest that this enhanced impact of facial color to our judgment of emotion may be attributable to biological and ecological factors.

## Author Contributions

TM, KN, and SN designed the study and wrote the initial draft of the manuscript. TM and KN contributed to analysis and interpretation of data. All authors approved the final version of the manuscript and agreed to be accountable for all aspects of the work in ensuring that questions related to the accuracy or integrity of any part of the work are appropriately investigated and resolved.

## Conflict of Interest Statement

The authors declare that the research was conducted in the absence of any commercial or financial relationships that could be construed as a potential conflict of interest.

## Footnotes

1 http://meesoft.logicnet.dk/
